# *Dolycoris baccarum* (L.) (Hemiptera: Pentatomidae) Infestation Induced Differential Changes in Plant Physiology: Preference and Damaging Nature to Different Sesame (*Sesamum indicum* L.) Varieties

**DOI:** 10.3390/insects17101001

**Published:** 2026-09-28

**Authors:** Seo Yeon Hong, Rameswor Maharjan, Jun Hyoung Jeon, Hwi Jong Yi, Young Nam Yoon, Ok Jae Won, Rahel Dinsa Guta, Jeong Eun Lee, Sung Up Kim

**Affiliations:** 1 Smart Agricultural Technology Division, Department of Upland Crop Research & Development, National Institute of Crop Science, Rural Development Administration, Miryang 50424, Republic of Korea; agriculture63@korea.kr (S.Y.H.); wjs258@korea.kr (J.H.J.); windsoul@korea.kr (H.J.Y.); yoonyn@korea.kr (Y.N.Y.); ojwon@korea.kr (O.J.W.); raheldinsa@korea.kr (R.D.G.); 2 Upland Crop Breeding Division, Department of Upland Crop Sciences, National Institute of Crop & Food Science, Rural Development Administration, Miryang 50424, Republic of Korea; jel6418@korea.kr (J.E.L.); sesameup@korea.kr (S.U.K.)

**Keywords:** shield bug, feeding behavior, photosynthesis, chlorophyll fluorescence signal, acid fuchsin test, relationships, management

## Abstract

*Dolycoris baccarum* (Hemiptera: Pentatomidae) is a cosmopolitan stink bug that causes substantial damage to several leguminous crops, including sesame. Herbivorous insect feeding can alter key physiological processes in plants, particularly photosynthesis and chlorophyll fluorescence, thereby affecting plant growth and productivity. This study investigated the effects of *D. baccarum* feeding on photosynthetic traits, including carbon assimilation rate (*A*) and stomatal conductance to water vapor (*g_sw_*), as well as chlorophyll fluorescence signal in different sesame varieties using a photosynthesis system and a portable gas exchange and fluorescence analyzer. Preference of *D. baccarum* among sesame varieties was evaluated through a choice test using seed pods, and feeding damage was quantified using the acid fuchsin test. Feeding by *D. baccarum* significantly reduced photosynthetic performance and chlorophyll fluorescence signal in sesame plants. The insect exhibited clear varietal preferences, and feeding damage differed significantly among sesame varieties. A positive association was observed between feeding activity and damage intensity, whereas photosynthetic parameters (*A* and *g_sw_*, and chlorophyll fluorescence signal) showed negative associations with yield following insect infestation. These findings indicate that *D. baccarum* feeding adversely affects both the physiological performance and productivity of sesame and highlight the potential for identifying resistant or tolerant sesame varieties for breeding programs. Further studies should examine the physical characteristics and nutrient composition of sesame seed pods and seeds to better understand the host selection behavior of *D. baccarum* and support the development of sesame varieties with improved resistance or tolerance to stink bug infestation.

## Simple Summary

The shield bug, *Dolycoris baccarum* (Hemiptera: Pentatomidae), is an important insect pest that threatens the production of several crops in Korea, particularly sesame (*Sesamum indicum* L.) and soybean (*Glycine max* L.). Delayed control measures can lead to substantial yield reduction and economic losses, highlighting the need for timely management. In this study, we investigated the effects of *D. baccarum* feeding on photosynthetic performance and chlorophyll fluorescence signal in different sesame varieties. In addition, laboratory experiments were conducted to evaluate varietal feeding preference and the extent of feeding damage caused by the insect. The results showed that *D. baccarum* feeding significantly altered photosynthetic activity and chlorophyll fluorescence characteristics. The insect also displayed distinct preferences among sesame varieties, and the severity of feeding damage varied markedly across genotypes. These findings provide important insights into the physiological mechanisms associated with sesame resistance and tolerance to *D. baccarum*. Identification of tolerant genotypes offers valuable genetic resources for breeding programs aimed at developing sesame cultivars with enhanced resistance or tolerance to stink bug infestation and supports the development of sustainable integrated pest management strategies.

## 1. Introduction

Stink bugs (Hemiptera: Pentatomidae) are among the most destructive piercing–sucking insect pests of agricultural crops worldwide and cause substantial economic losses in leguminous, cereal, and fruit crops in Korea [1,2]. Among these crops, soybean and sesame (*Sesamum indicum* L.) are particularly vulnerable to stink bug infestation. Both nymphs and adults feed preferentially on developing reproductive organs, apical meristematic tissues, and young leaves by inserting their stylets and injecting digestive enzymes, resulting in localized necrosis around feeding sites [3,4]. Such feeding injury reduces the seed quality, germination, and crop yield [1,5,6,7]. In addition to direct feeding damage, stink bugs can facilitate the transmission of viral, bacterial, and fungal pathogens [8]. In Korea, six stink bug species are recognized as major pests of field crops and fruit trees: *Riptortus pedestris*, *Piezodorus hybneri*, *Nezara antennata*, *Dolycoris baccarum*, *Halyomorpha halys*, and *Plautia stali* [9]. Among them, *D. baccarum* is widely distributed throughout Asia and Europe [10] and is highly polyphagous, feeding on numerous wild and cultivated plant species [11,12,13,14]. The species overwinters as diapausing adults, and its voltinism varies according to the regional temperature and photoperiod [12,15,16]. Despite its economic importance, the mechanisms underlying host plant preference and the physiological consequences of feeding by *D. baccarum* remain insufficiently understood.

Host plant selection is a fundamental determinant of herbivore survival, reproduction, and population dynamics. Female insects generally select host plants that maximize offspring survival and development [17,18,19,20]. Phytophagous insects discriminate among potential hosts based on their nutritional quality, defensive chemistry, physical characteristics such as trichome density, and interactions with natural enemies [21,22,23,24,25]. Plant volatile organic compounds are particularly important because they enable insects to detect and recognize suitable hosts over long distances [17,26,27,28]. Although many volatile compounds are shared among plant species, species-specific blends function as important host recognition cues [25,29]. Furthermore, previous experience with particular host plants can modify insect orientation, host acceptance, and oviposition behavior [30,31,32].

Herbivore feeding not only affects plant productivity through direct tissue damage but also disrupts photosynthetic processes. Chlorophyll, the primary pigment responsible for capturing light energy during photosynthesis, is essential for light absorption, energy transfer, and photochemical reactions [33,34]. Because chlorophyll content is positively correlated with photosynthetic capacity, reductions in chlorophyll often reflect impaired plant growth and productivity [35]. Feeding by sap-sucking insects frequently decreases the chlorophyll concentration by disrupting leaf tissues and reducing photosynthate availability [36,37]. Feeding and oviposition by the southern green stink bug, *Nezara viridula*, and *Murgantia histrionica* significantly reduced photosynthesis in French bean (*Phaseolus vulgaris*) and savoy cabbage (*Brassica oleracea*) [38]. Similar declines in chlorophyll content have been reported following infestation by the sweetpotato whitefly, *Bemisia tabaci* [39,40], and numerous other piercing–sucking insect species [41,42,43,44,45,46,47].

In addition to chlorophyll content, chlorophyll fluorescence analysis provides a sensitive, rapid, and non-destructive method for evaluating photosynthetic performance under biotic and abiotic stress [48]. One of the most informative fluorescence parameters is the effective quantum yield of photosystem II (ΦPSII), which is closely associated with carbon assimilation and photosynthetic electron transport [49,50]. Herbivore-induced damage frequently alters photosynthetic efficiency before visible symptoms become apparent. For example, feeding by larvae of *Trichoplusia ni* significantly reduced ΦPSII and CO_2_ assimilation in host plants [51,52]. Consequently, chlorophyll fluorescence has become an effective physiological indicator for quantifying the impact of insect herbivory on plant function.

Despite extensive studies [38,45,53,54,55] on the plant physiological responses of several plant species to herbivory for stink bug species, relatively little is known about how feeding by *D. baccarum* influences host plant photosynthetic performance and physiological status or whether these responses are associated with host suitability. Moreover, limited information is available on the impact of *D. baccarum* feeding on sesame in Korea. Understanding these interactions will improve knowledge of stink bug–plant relationships and may provide valuable physiological indicators for evaluating host susceptibility and developing integrated pest management strategies. In this study, we examined the sesame varieties preference of *D. baccarum* by observing bug performance on the selected sesame variety. We also measured the damage intensity and the physiological responses (photosynthesis and chlorophyll) of sesame varieties after feeding by *D. baccarum*.

## 2. Materials and Methods

### 2.1. Insect Collection and Rearing

*D. baccarum* adults were collected from sesame fields (Department of Upland Crop Research & Development, National Institute of Crop Science, Rural Development Administration (35°49′40′′ N, 128°74′01′′ E), Miryang, Gyeongnam Province, Korea) and reared on raw peanut and soybean seed along with sesame and soybean plants inside acrylic cages (40 × 40 × 40 cm, with side ventilation). Additionally, water was supplied in Petri dishes containing moistened cotton. After oviposition, the eggs attached on the plant leaves and stems were collected and transferred to Petri dishes for nymph hatching. Bugs were maintained under laboratory conditions (26 ± 1 °C, 60 ± 5% relative humidity, and a 16:8 h L:D photoperiod).

### 2.2. Sesame Plants

Sesame plants (cvs. SuperHani, YoungWoong, Suwon195, Yupung, Gangan, Haniall, Areum, Baegmi, Pyoungan, Koppom, Geonbaek, KangYou, Sangbaek, Suji, Chamhwang, Daheuk, Gangheuk, Ansan, Jobaek, Milsung, M80, M82, M83, M84, M86, and M87) were grown from seeds sown in plastic pots (18 cm diameter × 13 cm height) filled with a commercial soil medium (Baroker, Seoul Bio., Seoul, Republic of Korea). No chemical fertilizers or pesticides were applied. The plants were cultivated in a greenhouse maintained at 28 °C with a relative humidity of 60–80% under natural photoperiod conditions, and they were grown until seed pods had developed. The seeds of all tested sesame varieties were received from the Upland Crop Breeding Research Division, Department of Upland Crop Research & Development, National Institute of Crop Science, Rural Development Administration, Miryang, Korea (Table 1).

### 2.3. Insect Infestation

For the measurement of physiological responses, including photosynthesis and chlorophyll fluorescence imaging, adult bug infestations were conducted under controlled laboratory conditions (28 ± 1 °C, 60 ± 5% relative humidity, and a 16:8 h light:dark photoperiod). A single potted plant of each variety, aged 30–40 days after transplanting (DAT) and approximately 40–50 cm in height, was placed in a cage (2.5 m × 2.5 m × 2.5 m). A total of 100 mixed-sex adult bugs were released at the center of the plant arrangement. To minimize directional effects, the positions of the varieties were rotated clockwise daily. The insects were allowed to feed on the plants for 7 days, after which they were removed from the cages. The plants were then maintained under the same laboratory conditions until physiological measurements were performed.

### 2.4. Physiological Response Experiment

Photosynthesis: Photosynthetic parameters were measured using a portable photosynthesis system (LI-6800, LI-COR Corporation, 4647 Superior Street, P.O. Box 4425, Lincoln, NE 68504, USA). Measurements were taken from two leaves per sesame variety, with three replicates. Each pot with a sesame plant was considered a replicate, and the leaves were subsamples. During the measurements, the carbon assimilation rate (*A*) and stomatal conductance to water vapor (*g_sw_*) were recorded.

Chlorophyll fluorescence imaging: Chlorophyll fluorescence signal was assessed using a portable gas exchange and fluorescence system (GFS-3000 coupled with DE/PAM-2500, Heinz Walz GmbH, Eichenring 6, 91,090 Effeltrich, Germany). During the measurement of the chlorophyll fluorescence signal, we first calibrated the device with *F_v_*/*F_m_* and then measured the effective quantum yield of photosystem Y(II). Two leaves from each sesame variety were measured, and the experiment was repeated three times. Each pot with a sesame plant was considered a replicate.

Both photosynthetic and chlorophyll fluorescence signal measurements were conducted on sesame plants before bug infestation and again after infestation to evaluate the physiological effects of insect feeding. The physiological comparisons in this study are labeled as exploratory because the experimental design involves pseudoreplication (Supplementary Table S1).

### 2.5. Preference Experiment

The preference of bugs for sesame green pod seeds was evaluated under controlled laboratory conditions (28 ± 1 °C, 60 ± 5% relative humidity, and a 16:8 h light:dark photoperiod). Four green seed pods from each sesame variety were placed in individual Petri dishes (5 cm diameter × 1.5 cm height), and all dishes were arranged inside an acrylic cage (85 cm × 55 cm × 55 cm) that served as the experimental arena. After the arena was prepared, 100 adult bugs that had been starved for 24 h were released at the center of the cage. To minimize directional bias, the positions of the Petri dishes were rotated clockwise each day. The bug preference was assessed by counting the number of insects present on each sesame variety, including individuals who were either resting or feeding. During the observation period, if insects were just staying without feeding, this was considered a staying activity; when insects were staying and sucking seed pods, this was considered a feeding activity. Observations were recorded at 2 h intervals from 9:00 AM to 6:00 PM for three consecutive days. The experiment was conducted with three replications.

### 2.6. Yield Assessment

The yield of sesame varieties was assessed at the harvesting stage (dried seed pod condition). Once the dried seed pods per plant were harvested, the seeds were extracted from pods and then dried on the shed at ambient temperature for 48 h. The weight (gm) of the dried seeds was then measured with a digital balance (Sartorius, CP1245, Göttingen, Lower Saxony, Germany). The assessment was repeated three times.

### 2.7. Damage Assessment

Feeding damage caused by bugs was further evaluated using the acid fuchsin test, a method commonly employed to detect insect feeding injury on seeds [56,57,58]. For the experiment, matured pod seeds were harvested from the sesame varieties exposed to *D. baccarum* adults. Sesame seeds were removed from harvested pods and immersed for 1 h in an acid fuchsin staining solution consisting of 1 g acid fuchsin, 250 mL 95% ethanol, and 250 mL acetic acid. This staining procedure enabled visualization of the stylet sheaths left by feeding insects. After staining, the pods were rinsed thoroughly with running tap water, and the number of stylet sheaths was counted under magnification using a stereomicroscope (LEICA M125 microscope and imaging system, Germany) [59,60] and categorized into three damage levels (Figure 1). The damage percentage was calculated by dividing the damaged number of seeds by the total number of seeds and then multiplying by 100.

### 2.8. Statistical Analysis

The data obtained for all measured parameters were subjected to statistical analysis whenever appropriate. Differences among sesame varieties for host choice, photosynthetic traits, chlorophyll fluorescence signal, and damage assessment were evaluated using one-way analysis of variance (ANOVA), and differences among sesame varieties for relative feeding activity was analyzed using repeated-measures ANOVA with the procedure (PROC GLM). Tukey’s studentized range honestly significant difference (HSD) was performed at *p* < 0.05 level of significance for the treatment significant test. The relationships between feeding activity and damage rate and between yield and photosynthesis and chlorophyll fluorescence were examined using the general linear model (GLM) to determine any potential association between these two variables. All statistical analyses were performed using SAS software, ver. 9.4 [61].

## 3. Results

### 3.1. Physiological Response Experiment

After *D. baccurum* feeding, the photosynthesis parameters, the carbon assimilation rate (*A*) (F = 7.99, df = 25,130, *p* < 0.0001) and stomatal conductance to water vapor (*g_sw_*) (F = 2.87, df = 25,130, *p* < 0.0001) significantly changed among the sesame varieties. The highest reduction in A was recorded on SuperHani (87.68%), followed by Pyoungan (84.62%) and Jobaek (74.08%). The lowest change was (19.55%) for the Haniol variety. However, the carbon assimilation rate (*A*) was found to increase for the M84, M86, and M87 varieties (Figure 2a).

In the case of *g_sw_*, the highest change in *g_sw_* was recorded on Suwon195 (89.81%), followed by Jobaek (80.35%) and Pyoungan (67.91%). The lowest reduction (4.92%) was for the YoungWoong variety. However, gsw was found to increase for the Gangan, KangYou, Sangbaek, M80, M84, M86, and M87 varieties (Figure 2b).

The chlorophyll fluorescence parameter Y(II)) after *D. baccurum* feeding was also significantly varied among sesame varieties (F = 4.58, df = 25,52, *p* < 0.0001). The highest change in the chlorophyll fluorescence signal was recorded on Pyoungan (17.39%), followed by Jobaek (8.47%) and YoungWoon (6.50%). The lowest change (0.06%) was for the M80 variety. However, the chlorophyll fluorescence signal was found to increase for the Baegmi, Kopoom, Ansan, and Milsung varieties (Figure 3).

### 3.2. Preference Experiment

*Dolycoris baccarum* adults showed significantly different preferences toward sesame varieties, with 88.00% of tested bugs demonstrating a preference for Chamhwang, followed by Pyoungan (50.00%) and Kopoom (50.00%) in the laboratory experiment (F = 7.17, df = 25,52, *p* < 0.0001). Staying and feeding activities were also found to differ among sesame varieties (Figure 4). Bugs had a higher staying rate (34.00%) on Pyoungan, followed by Kopoom (31.30%) and KangYou (29.30%) (F = 3.35, df = 22,216, *p* < 0.0001). A higher feeding rate (64.00%) was recorded on Chamhwang, followed by Kopoom (18.70%), and the lowest feeding rate (0.7%) was on Milsung (F = 3.50, df = 22,153, *p* < 0.0001) (Figure 5).

### 3.3. Damage Assessment

The acid fuchsin test results show that the sesame seed damage rate was significantly different among varieties (F = 10.54, df = 21,44, *p* < 0.0001). Higher seed damage (18.52%) was recorded on Chamhwang, followed by Suwon195 (17.78%) and Suji (14.81%), the lowest seed damage was recorded on Areum (3.00%), and no seed damage was observed on YoungWoong (Figure 6A).

In the case of multiple seed damage, it was also significantly different among varieties (F = 4.98, df = 21,44, *p* < 0.0001). Higher multiple seed damage (19.00%) was recorded on Chamhwang, followed by SuperHani (15.56%) and Suji (11.11%), the lowest (2.22%) multiple seed damage was recorded on Areum, and no multiple seed damaged was observed on YoungWoong and M80 (Figure 6B).

In the case of deep seed damage, it was also significantly different among varieties (F = 2.79, df = 21,44, *p* = 0.0021). Higher deep seed damage (11.21%) was recorded on Chamhwang, followed by Suwon195 (8.89%) and SuperHani (5.56%), the lowest (1.11%) deep seed damage was recorded on Gangan, Areum, Pyoungan, and Kopoom, respectively, and no deep seed damage was observed on Gangheuk, Ansan, YoungWoong, Jobaek, Milsung and M82 (Figure 6C).

### 3.4. Relationships

There was a positive relationship between the feeding activity and the seed damage rate (R^2^ = 0.6132; Reg. F = 7.10, df = 1,21, *p* = 0.0145) (Figure 7). Similarly, Table 2 shows the relationships between yield and photosynthesis (A and gsw) and the chlorophyll fluorescence signal. The sesame yield was negatively correlated with photosynthesis (*A*: F = 18.67, df = 1,22, *p* = 0.0003; *g_sw_*: F = 10.45, df = 1,20, *p* = 0.0042) and the chlorophyll fluorescence signal (F = 6.25, df = 1,20, *p* = 0.0213) (Table 2).

## 4. Discussion

Photosynthesis and chlorophyll fluorescence signal are fundamental physiological processes that support plant growth, development, and productivity by converting light energy into chemical energy required for metabolic activities [62]. Because these processes are central to plant function, they are highly sensitive to biotic stresses, including herbivore infestation [63]. Herbivore feeding can disrupt photosynthetic mechanisms and reduce photosynthetic efficiency, resulting in substantial physiological damage to plants [63,64,65]. In the present study, *D*. *baccarum* infestation reduced photosynthetic parameters, particularly the carbon assimilation rate (A) and stomatal conductance (gsw), as well as chlorophyll fluorescence signal in most sesame varieties. These findings are consistent with previous studies showing that insect pests such as *Nezara viridula*, *Murgantia histrionica*, *Stephanitis pyrioides*, *Bagrada hilaris*, *Apolygus lucorum*, *Lycorma delicatula*, whiteflies, and aphids can significantly impair photosynthetic processes [38,39,40,45,66,67,68,69]. Similar reductions in photosynthesis have also been reported under pathogen infection [70,71,72], emphasizing the vulnerability of the photosynthetic apparatus to biotic stress. Furthermore, a meta-analysis by [73] demonstrated that sap-feeding insects generally reduce plant growth and photosynthesis, with generalist herbivores often causing stronger negative effects than specialists.

Chlorophyll fluorescence is the primary photosynthetic pigment responsible for light absorption, energy transfer, and photochemical reactions [33,34]. Because chlorophyll fluorescence signal is positively correlated with photosynthetic capacity, changes in chlorophyll provide a useful indicator of plant physiological status [35]. In this study, chlorophyll declined markedly in sesame plants exposed to *D. baccarum*. Similar reductions in chlorophyll have been reported in herbaceous plants subjected to feeding and oviposition by *M. histrionica* and *N. viridula* [38]. Likewise, Ref. [74] observed significant decreases in chlorophyll a and b contents and associated photosynthetic parameters following *A. lucorum* infestation. Herbivore damage is known to alter primary photosynthetic reactions, and fluorescence imaging studies have shown that feeding by *Trichoplusia nilarvae* reduces the quantum yield of photosystem II and CO_2_ uptake in host plants [51,52]. Suppression of gas exchange by *Lycorma delicatula* feeding has also been documented in maple saplings [75]. These studies support the conclusion that *D. baccarum* feeding disrupts photosynthetic performance in sesame.

The magnitude of the reduction in photosynthesis and chlorophyll fluorescence signal differed among sesame varieties, suggesting substantial varietal variation in physiological responses. Such variation may be associated with morphological traits, including the leaf tissue structure, wax deposition, trichome density, and varietal growth rate, which can influence insect attraction or deterrence [76,77,78]. Genetic factors are also likely involved. Herbivore feeding has been shown to alter the expression of photosynthesis-related genes [79,80,81,82,83]. Ref. [74] further demonstrated that chlorophyll biosynthesis and photosynthesis involve complex metabolic pathways regulated by numerous genes, and disruption of these pathways can substantially reduce chlorophyll production [84,85]. Therefore, the varietal differences observed in this study may reflect differences in the genetic regulation of photosynthetic and chlorophyll biosynthetic pathways during herbivore attack.

Interestingly, some sesame varieties maintained or even increased photosynthetic activity after *D. baccarum* infestation. For example, the increase in the carbon assimilation rate (*A*) in M84, M86, and M87 and the chlorophyll fluorescence signal in Baegmi, Kopoom, Ansan, and Milsung varieties may be due to enhanced photosynthesis attributed to reactive oxygen species (ROS) signaling and compensation. This also can be explained as a significant increase in steady-state fluorescence observed in certain varieties, given the concurrent decrease in *F_v_*/*F_m_* and the restricted induction of NPQ; this increase likely reflects a reduction in photochemical quenching (*q_p_*) stemming from photoinhibitory stress, rather than an increase in light-harvesting efficiency. Under these conditions, the closed PSII reaction centers are unable to process excitation energy efficiently, leading to the observed rise in steady-state fluorescence. While this state often correlates with heightened oxidative stress, the exact downstream responses remain to be fully elucidated. We hypothesize that the plant may initiate ROS-mediated signaling or alternative electron transport pathways as a temporary compensation mechanism to prevent runway photo-oxidation. However, further analyses are required to confirm these specific target pathways [86]. These tolerant varieties may either sustain photosynthetic capacity during feeding or recover photosynthetic function more rapidly after insect removal through compensatory responses. Ref. [87] reported that feeding by *Spodoptera exigua* for 3 h did not reduce whole-leaf photosystem II efficiency, indicating effective compensation with minimal photosynthetic cost. Similarly, Ref. [69] observed rapid ROS generation after herbivore attack, which was considered an important early defense mechanism. The present findings suggest that some sesame genotypes possess enhanced physiological resilience to *D. baccarum* feeding.

The host preference assays showed that *D. baccarum* consistently preferred certain sesame varieties, particularly Chamhwang, Kopoom, and Pyoungan. The insects spent less time searching and more time feeding on these varieties, indicating that olfactory cues play an important role in host location and acceptance. Similar host-selection behavior has been reported in other hemipteran insects. Preference may be influenced by seed and pod characteristics, including the size, shape, color, seed coat texture, hardness, moisture content, and chemical composition [88,89,90]. Structural barriers such as trichomes and hard seed coats can impede stylet penetration and reduce the feeding efficiency, especially in immature stages [89]. In addition, allelochemicals and other secondary metabolites can influence feeding and oviposition behavior [91]. The strong preference for Chamhwang, Kopoom, and Pyoungan suggests that these varieties possess physical or chemical traits that favor feeding by *D. baccarum*.

Acid fuchsin staining further revealed significant varietal differences in feeding damage, with Chamhwang, Suwon195, and Suji exhibiting the highest damage levels. As with host preference, these differences may be associated with the pod and seed physical properties and chemical composition. Previous studies demonstrated that seed coat characteristics strongly affect the penetration, survival, and development of seed-feeding insects such as *Callosobruchus maculatus* [92,93,94]. Although nutrient composition was not analyzed in the present study, it is plausible that the preferred and highly damaged varieties provide more suitable nutritional resources for *D. baccarum* development.

General linear module analyses showed positive associations between the feeding activity and damage level and negative associations between the sesame yield and photosynthetic traits and the chlorophyll fluorescence signal. These relationships indicate that increased bug feeding intensifies tissue damage, reduces photosynthetic performance and chlorophyll status, and ultimately contributes to yield reduction. Thus, physiological impairment appears to be an important mechanism linking insect infestation to productivity loss in sesame. However, a key limitation of detached-pod assays for estimating crop damage is that the responses may not accurately reflect the damage to intact plants because detachment alters physiological defenses, nutritional characteristics, and plant compensatory mechanisms. Although detached-pod assays are widely used to screen pest and disease resistance, they lack the systemic and physiological context of living plants and should therefore be interpreted with caution.

Overall, the present study implies that *D. baccarum* infestation adversely affects photosynthesis and chlorophyll fluorescence signal in sesame, although the severity of damage varies considerably among varieties. The maintenance of photosynthetic activity in some genotypes suggests the presence of tolerant lines with enhanced physiological resilience to bug feeding. These findings provide important insight into the physiological basis of sesame resistance and tolerance to *D. baccarum* and may contribute to breeding programs aimed at developing sesame varieties with improved tolerance to stink bug infestation. Despite this physiological basis, the observed difference responses may also be associated with infestation rather than definitive evidence of causal effects or varietal tolerance. Moreover, future studies should use independently replicated infested and non-infested plants for each sesame variety to provide stronger evidence of the physiological responses and tolerance.

## 5. Conclusions

The present study demonstrated that *D*. *baccarum* infestation significantly affects the physiological performance of sesame by reducing photosynthetic parameters, particularly the carbon assimilation rate (A) and stomatal conductance (gsw), as well as the chlorophyll fluorescence signal in most tested varieties. These findings suggest that stink bug feeding disrupts photosynthetic processes and chlorophyll-related functions, leading to physiological stress that can contribute to yield reduction. Significant varietal differences were observed in the photosynthetic response, chlorophyll fluorescence signal, host preference, and feeding damage, indicating that sesame genotypes differ markedly in their susceptibility to *D. baccarum*. Some sesame varieties maintained relatively stable photosynthetic activity following infestation, suggesting the presence of tolerance mechanisms associated with enhanced physiological resilience and defense responses. The observed positive relationship between feeding activity and damage, together with the negative relationship between yield and photosynthetic traits and the chlorophyll fluorescence signal indicates that bug infestation along with physiological impairment are major mechanisms underlying productivity loss in sesame. Overall, this study provides valuable insight into the physiological basis of sesame resistance and tolerance to *D. baccarum*. The identification of tolerant genotypes offers a useful foundation for breeding programs aimed at developing sesame varieties with improved resistance or tolerance to stink bug infestation and for designing sustainable integrated pest management strategies.

## Figures and Tables

**Figure 1:**
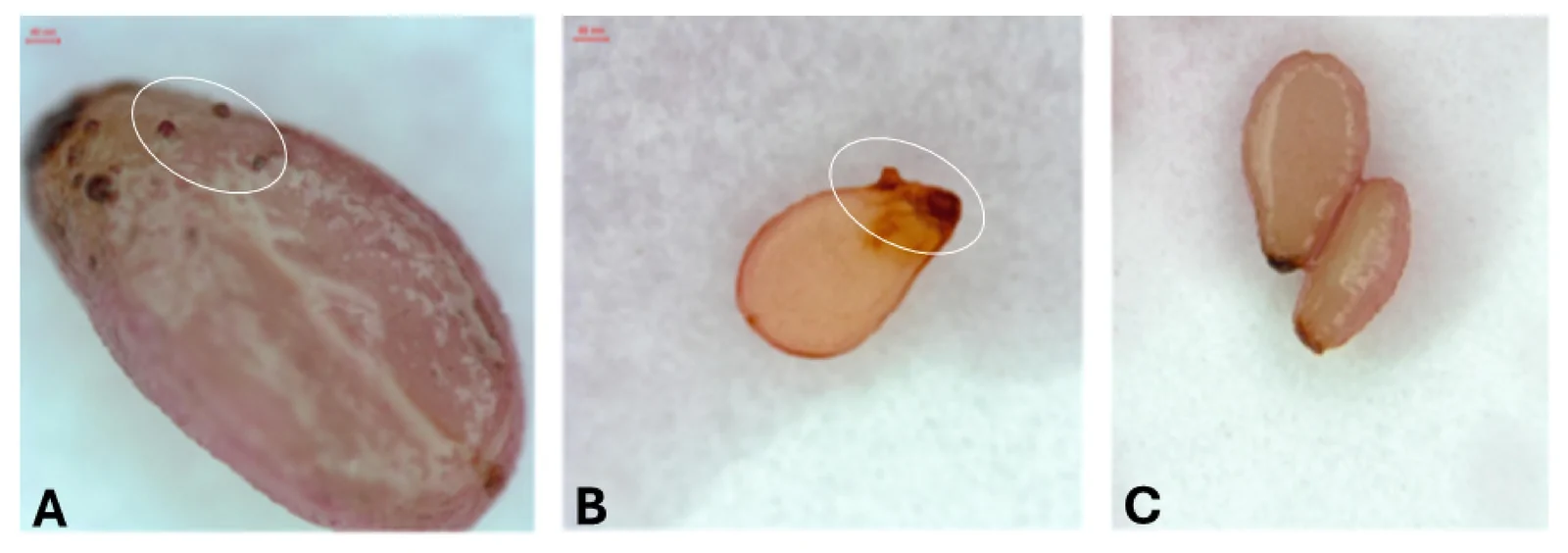
Damage characteristics of sesame seeds detected via acid fuchsin test after feeding by *Dolycoris baccarum*. Seed damage was categorized into (**A**) multiple damage, (**B**) deep damage, and (**C**) non-damaged based on the size and no. of stylet sheaths (marked with white circle) present on the seeds. Multiple damage was categorized as presence of more than one stylet sheath; deep damage was categorized as presence of larger stylet sheath, and non-damaged was categorized as absence of any stylet sheaths on the seeds. Seeds with stylet sheaths were photographed at 40 nm with a stereomicroscope.

**Figure 2:**
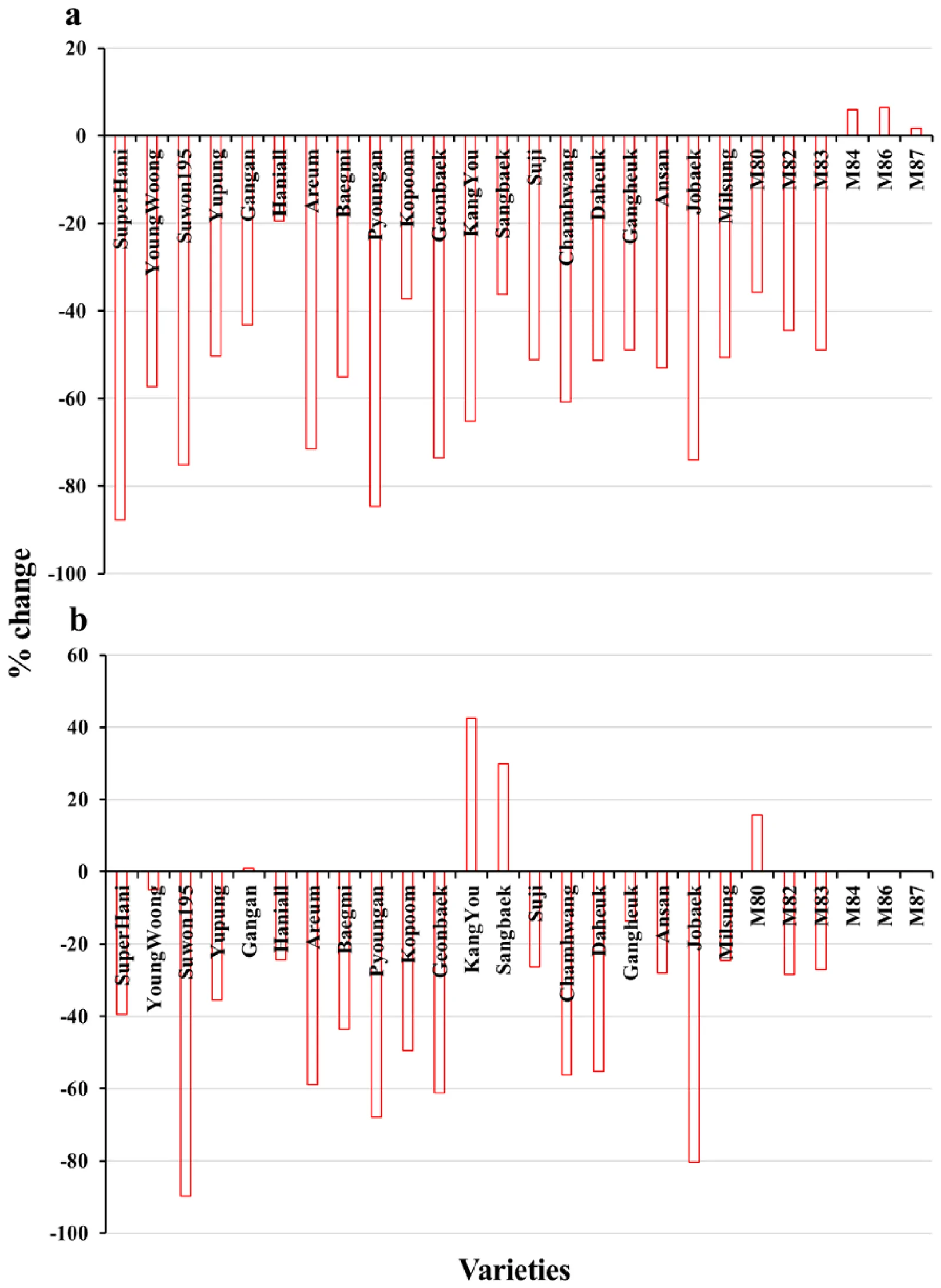
Percent change of (**a**) the carbon assimilation rate (*A*) and (**b**) stomatal conductance to water vapor (*g_sw_*) in the photosynthesis process after infestation of *Dolycoris baccarum* for a week.

**Figure 3:**
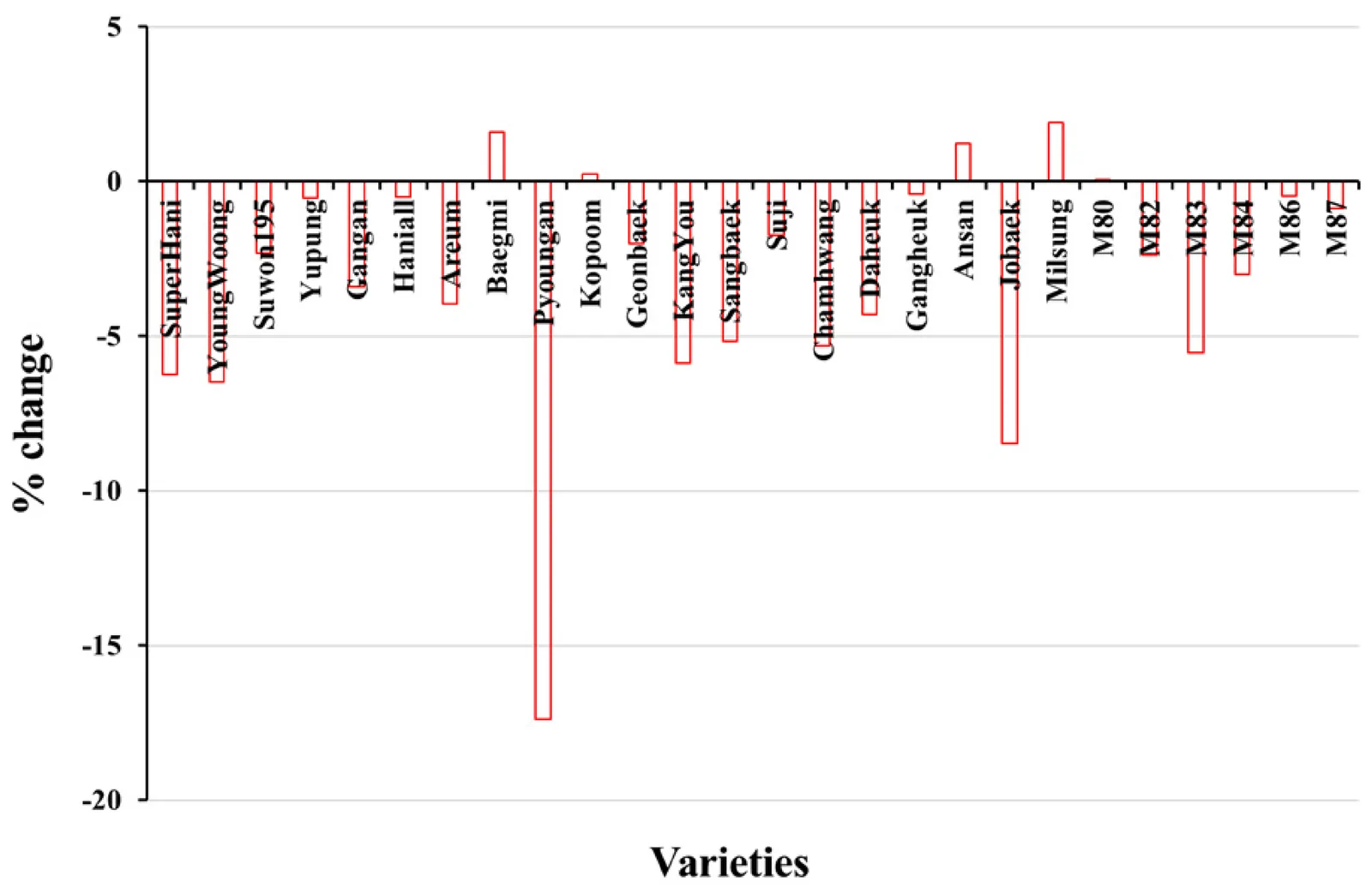
Percent change of chlorophyll fluorescence signal in the sesame varieties after infestation of *Dolycoris baccarum* for a week.

**Figure 4:**
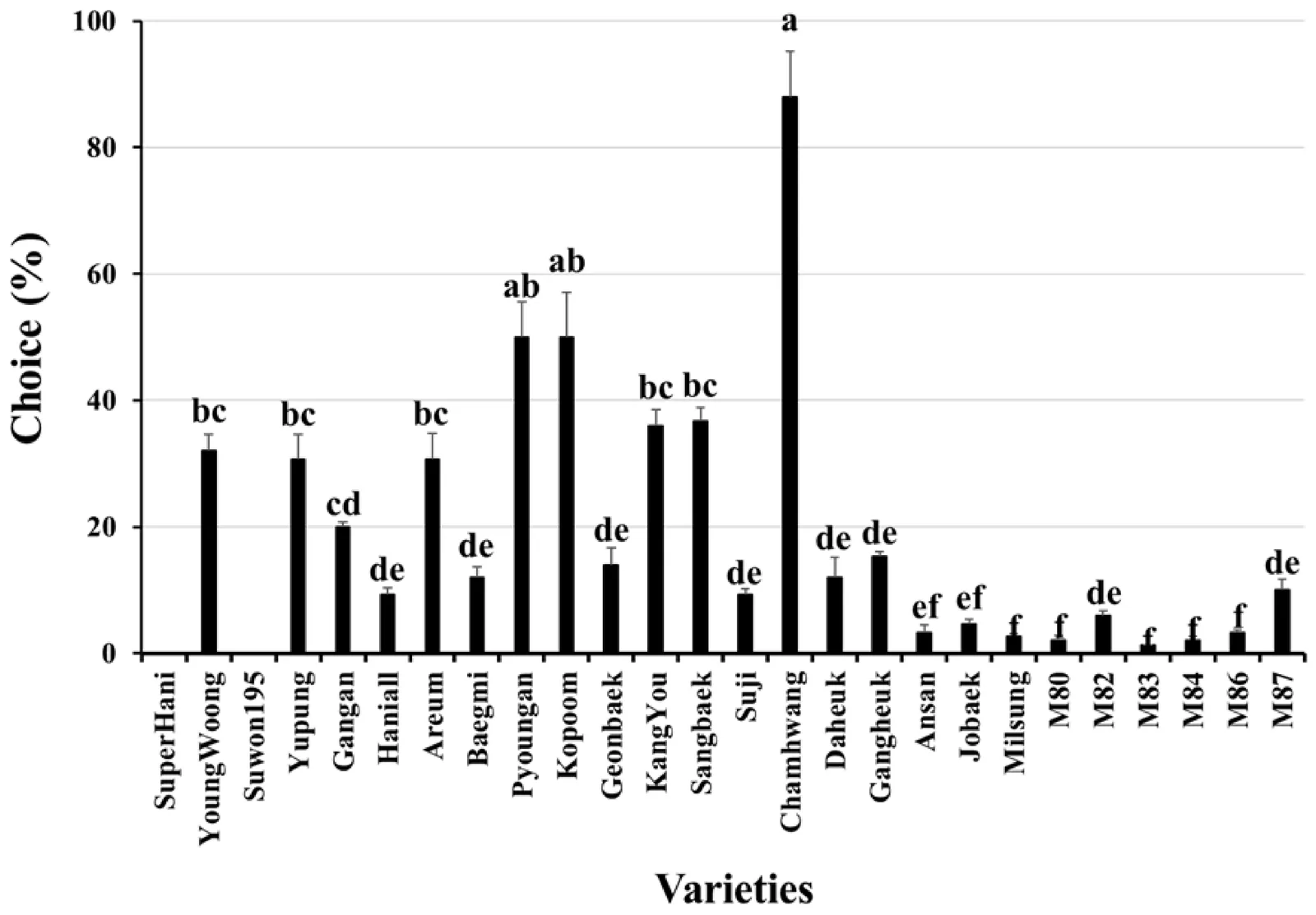
Choice of *Dolycoris baccarum* on different sesame varieties in the laboratory experiment. Bars represent standard errors. The same letters above the bar denote no significant difference compared by Tukey’s studentized range honestly significant difference (HSD) test (ANOVA, *p* < 0.05).

**Figure 5:**
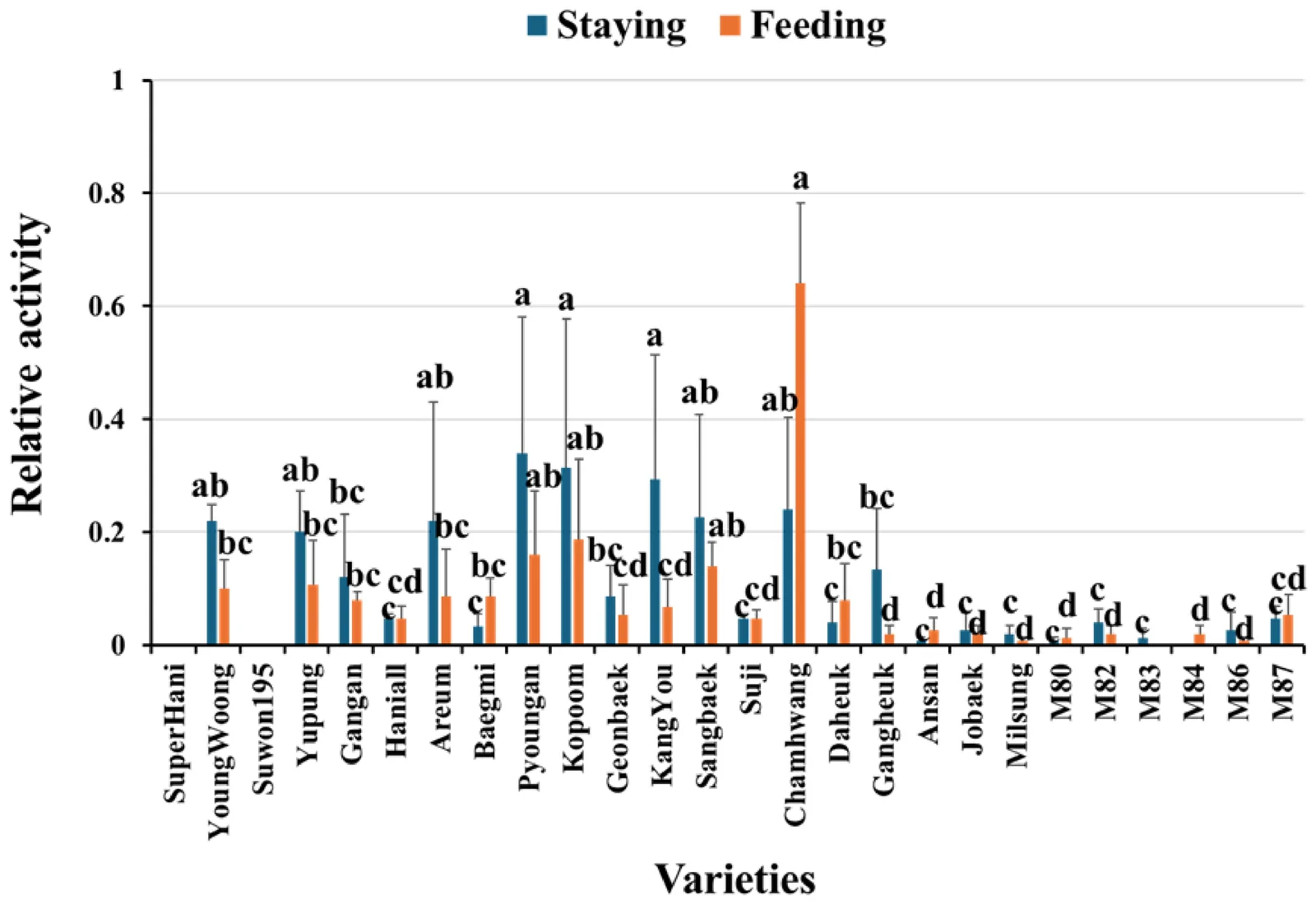
Relative activity of staying and feeding activity of *Dolycoris baccarum* on different sesame varieties in laboratory experiment. Bars represent standard errors. The same letters above the bar denote no significant difference compared by Tukey’s studentized range honestly significant difference (HSD) test (ANOVA, *p* < 0.05).

**Figure 6:**
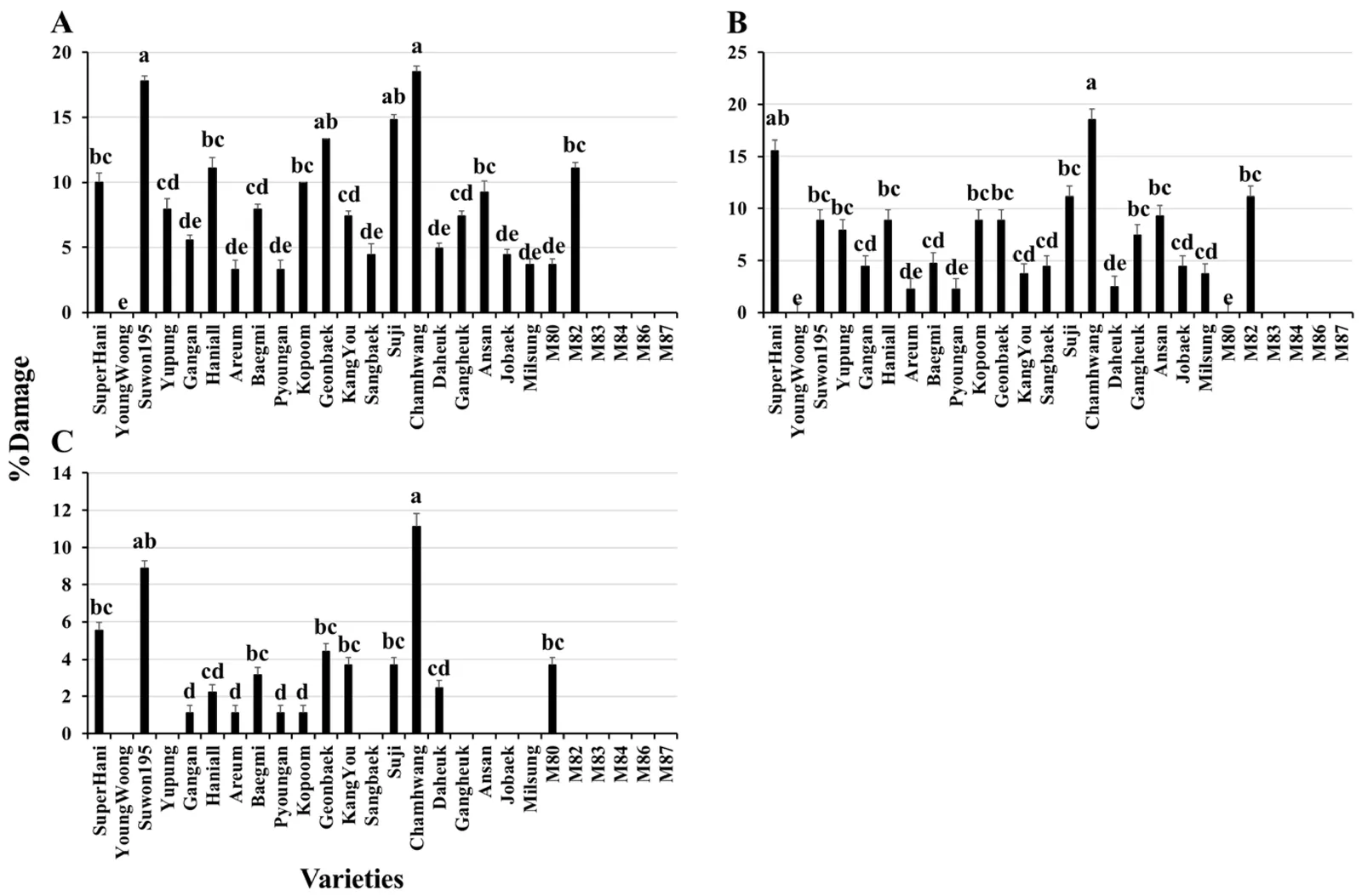
Damage assessment, over all seed damage (**A**), multiple seed damage (**B**), and deep seed damage (**C**) in sesame varieties after feeding by *Dolycoris baccarum* with acid fuchsin test. Damage was categorized based on the size of lesions on the seeds after sucking via AFT. Data for M83, M84, M86 and M87 are not shown due to low quality of seeds. Bars represent standard errors. The same letters above the bar denote no significant difference compared by Tukey’s studentized range honestly significant difference (HSD) test (ANOVA, *p* < 0.05).

**Figure 7:**
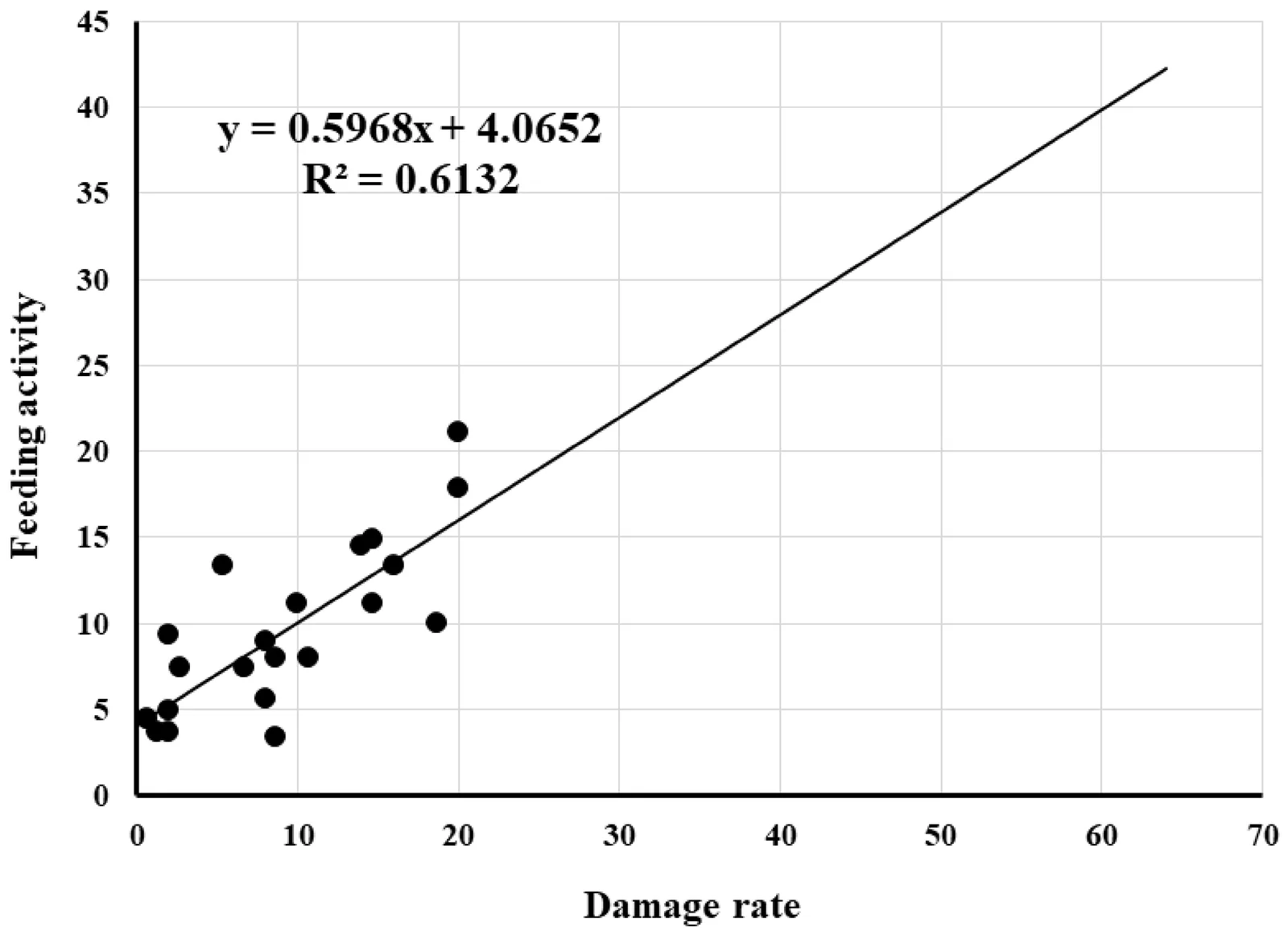
Relationships between feeding activity and damage rate of *Dolycoris baccarum* (y = 0.5968x + 4.0652; R^2^ = 0.6132; *p* = 0.0145; regression significant at *p* < 0.05), 3 days after seed pod exposure to *Dolycorius baccarum* in the laboratory. One hundred adult bugs were released. Feeding activity in this figure denotes the seed sucking state; data were used from 26 sesame varieties.

**Table 1:** Varietal breeding period and characteristics (maturity, seed and flower color) of 26 sesame cultivars used in the experiment with each pedigree.

Variety	Breeding Year	Maturity Type	Seed Coat Color	Flower Color	Pedigree
SuperHani	2022	Late	White	Pink	Suwon195(IT345437)/YSC71
YoungWoong	2023	Medium	White	White	IT326979/γ-ray 300gy
Suwon195	na	Medium	White	White	S8826/SI90033-14//SB764
Yupung	2010	Medium	Brown	White	Traditional variety
Gangan	2014	Medium	White	White	Hwangsan(IT229440)/Ahnsan(IT030433)
Haniall	2021	Early	White	White	Suwon195(IT345437)/IT030100
Areum	2010	Late	Black	White	IT311646/IT229438
Baegmi	na	Early	White	White	na
Pyoungan	2006	Medium	White	White	Ahnsan(IT030433)/X-ray 200Gy
Kopoom	1994	Medium	White	White	Dongro/SC645//Meteor/Mokpo no.9
Geonbaek	2013	Medium	White	White	Seongbunkkae/SIG950006-4-1-1
KangYou	2019	Medium	White	White	China Black/SI982849
Sangbaek	2011	Medium	White	Pink	Suwon195(IT345437)/Yangbaek(IT229436)
Suji	2010	Medium	White	White	Traditional variety
Chamhwang	1999	Medium	Brown	White	(IT311646)/S96
Daheuk	2009	Early	Black	White	(IT362712)//IT030511)/(IT229439)
Gangheuk	2003	Medium	Black	White	S8826/S190033-14//SB764
Ansan	1985	Early	White	White	IT030100 Radiation breeding
Jobaek	2018	Early	White	White	Ahnsan(IT030433)/Suwon no.178
Milsung	2007	Medium	White	Pink	Iri no. 4/Suwon no. 131
M80	br	-	-	-	-
M82	br	-	-	-	-
M83	br	-	-	-	-
M84	br	Medium	Black	White	Gangan(IT314750)/Gangheuk(IT221525)
M86	br	Late	Black	White	Areum/Suwon195(IT345437)
M87	br	Medium	White	White	Geonbaek(IT326979)/Gangan(IT314750)

Note: br—breeding continues; na—not available. Source: Rural Development Administration, Korea.

**Table 2:** Relationships between yield (gm) and physiological responses, photosynthesis parameters, the carbon assimilation rate (*A*) (*μ*mol CO_2_ m^−2^ s^−1^) and stomatal conductance to water vapor (*g_sw_*) (mol H_2_O m^−2^ s^−1^) and chlorophyll fluorescence signal (Y(II)) of sesame varieties.

Parameter	Physiological Responses	Linear Equation	R^2^	*p*-Value
Yield	Carbon assimilation rate (*A*)	y = −42.107x + 102	0.459	0.0003
	Stomatal conductance to water vapor (*g_sw_*)	y = −40.168x + 91.283	0.3432	0.0042
	Chlorophyll fluorescence signal (Y(II))	y = −16.347x + 32.999	0.2793	0.0213

Regression significant at *p* < 0.05.

## Data Availability

Data are available upon request from the corresponding author of this manuscript due to official rules and norms.

## Supplementary Materials

The following supporting information can be downloaded at https://www.mdpi.com/article/10.3390/insects17101001/s1, Table S1: Change in photosynthesis parameters after *Dolycoris baccarum* infestation on different sesame varieties; Table S2: Change in chlorophyll flurosecnce signal after *Dolycoris baccarum* infestation on different sesame varieties.
